# The Strength of hERG Inhibition by Erythromycin at Different Temperatures Might Be Due to Its Interacting Features with the Channels

**DOI:** 10.3390/molecules28135176

**Published:** 2023-07-03

**Authors:** Dongrong Cheng, Xiaofeng Wei, Yanting Zhang, Qian Zhang, Jianwei Xu, Jiaxin Yang, Junjie Yu, Antony Stalin, Huan Liu, Jintao Wang, Dian Zhong, Lanying Pan, Wei Zhao, Yuan Chen

**Affiliations:** 1 Chinese Herb Medicine Division, Zhejiang Agriculture and Forestry University, 666 Wusu Street, Hangzhou 311300, China; d.r.cheng@outlook.com (D.C.); 2020102131011@stu.zafu.edu.cn (X.W.); anran1378@gmail.com (Y.Z.); zqmztkn@163.com (Q.Z.); xujw1971@zafu.edu.cn (J.X.); yangjiaxin178@163.com (J.Y.); 17816898621@163.com (J.Y.); 2020602052004@stu.zafu.edu.cn (H.L.); 18275995437@163.com (J.W.); d.zhong@outlook.com (D.Z.); 2 The State Key Laboratory of Subtropical Silviculture, Zhejiang Agriculture and Forestry University, 666 Wusu St, Hangzhou 311300, China; 3 Institute of Fundamental and Frontier Sciences, University of Electronic Science and Technology of China, Chengdu 610064, China; antonystalin@uestc.edu.cn; 4 Shuren International Medical College, Zhejiang Shuren University, Hangzhou 310009, China

**Keywords:** erythromycin, temperature, hERG, steady-state activation, steady-state inactivation

## Abstract

Erythromycin is one of the few compounds that remarkably increase ether-a-go-go-related gene (hERG) inhibition from room temperature (RT) to physiological temperature (PT). Understanding how erythromycin inhibits the hERG could help us to decide which compounds are needed for further studies. The whole-cell patch clamp technique was used to investigate the effects of erythromycin on hERG channels at different temperatures. While erythromycin caused a concentration-dependent inhibition of cardiac hERG channels, it also shifted the steady-state activation and steady-state inactivation of the channel to the left and significantly accelerated the onset of inactivation at both temperatures, although temperature itself caused a profound change in the dynamics of hERG channels. Our data also suggest that the binding pattern to S6 of the channels changes at PT. In contrast, cisapride, a well-known hERG blocker whose inhibition is not affected by temperature, does not change its critical binding sites after the temperature is raised to PT. Our data suggest that erythromycin is unique and that the shift in hERG inhibition may not apply to other compounds.

## 1. Introduction

The human ether-a-go-go-related gene (hERG) is important for the cardiac action potential (AP). It is one of the critical components for the formation of the plateau of the cardiac AP and determines the duration of the QT interval in the electrocardiogram (ECG) as it plays an essential role in AP repolarization [1,2,3,4,5,6]. Thus, the inhibition of hERG channels will lead to a prolongation of the QT interval of the ECG, which is associated with a risk for the potentially fatal arrhythmia torsades de pointes (TdP) [7,8].

The hERG channel is a tetramer consisting of four pore-forming α-subunits [9]. It has a unique pore structure with a small central cavity with extended pockets that promote the binding of cationic drugs [10] (Wang et al., 2017). The extended pockets also have properties that favor drug binding as they are elongated and relatively hydrophobic. In addition, the pore region also contains two aromatic amino acid residues, Y652 and F656, in the S6 helices. These unique properties are thought to contribute to the sensitivity of hERG to various pharmacological blockades [7,11,12,13,14]. 

Because there is a clear association between hERG channel inhibition and many non-antiarrhythmic drugs that produce torsades de pointes associated with QT prolongation, an early assessment of hERG toxicity is strongly recommended by regulatory agencies such as the U.S. Food and Drug Administration (FDA) and the European Medicines Agency (EMA). Such assessments are usually performed at room temperature (RT), which is different from the physiological temperature of 37 °C (PT). A few compounds such as erythromycin and sotalol have a much stronger inhibition at physiological temperature [15,16]. Although the concept of comprehensive in vitro proarrhythmic assay (CiPA) testing was developed to ensure cardiac safety [17] (Fermini et al., 2016), hERG screening is still a major concern for cardiac safety. 

It is a concern when compounds are screened against hERG only at RT. However, due to a higher metabolism and oxygen demand, it might be much more difficult to screen PT using the whole-cell patch clamp technique. Therefore, it is very time consuming and could be a waste of money to screen all these compounds at PT. Thus, it is important to understand when compounds need to be further investigated at physiological temperature.

In the early stages of drug discovery, the goal is to find a lead compound, i.e., a chemical compound that has pharmacological or biological activity, that is likely to be therapeutically useful but has a suboptimal structure that needs further modification. Therefore, it is important to understand how reliably compounds are investigated at room temperature.

Erythromycin, a macrolide antibiotic drug, is of interest because it is one of the few compounds that exhibit an approximately 10-fold difference in hERG inhibition at different temperatures [15,18]. Erythromycin has also been reported to have a protective effect against hERG blockade [19,20]. However, these studies either did not investigate the mechanism or did not use a commercial device for precise temperature control. Characterizing the behavior of erythromycin against hERG at RT and PT will help us to understand whether a compound has multiple folds of IC_50_ of hERG inhibition at these two temperatures and whether it is necessary to screen it against hERG channels in early drug development. 

## 2. Results 

### 2.1. Temperature-Dependent Inhibition of hERG by Erythromycin

**Figure 1:**
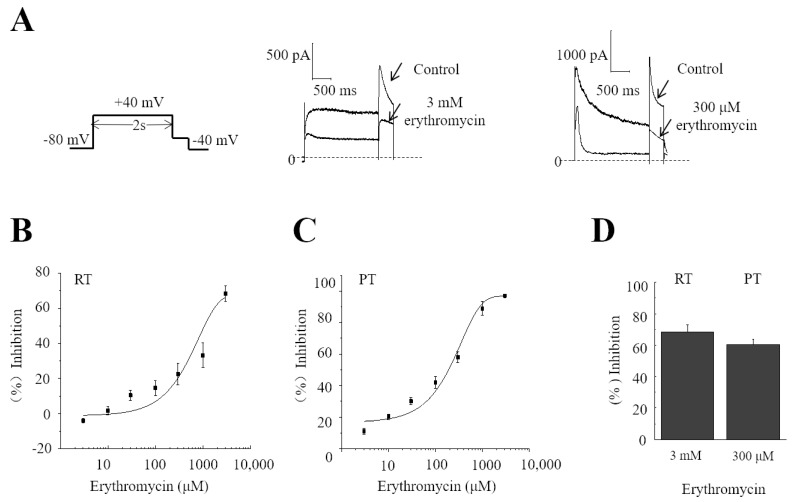
The percentage inhibition of erythromycin on hERG currents at room temperature (RT) and physiological temperature (PT). (**A**) The left panel shows the command voltage protocol. The middle and right panels show the example current traces at RT and at PT. (**B**) The concentration response curve shows the inhibition of erythromycin on hERG currents at different concentrations at RT (*n* = 5–10). Data were fitted using the Boltzmann function Hill equation. (**C**) Different concentrations of erythromycin on hERG currents at PT (*n* = 7). (**D**) The average percentage inhibition rates of 3 mM erythromycin at RT (*n* = 5) and 300 µM at PT (*n* = 7) on hERG channels.

In this series of experiments, different concentrations of erythromycin were used to investigate the response of hERG channels at RT and at PT, respectively. The protocol is shown in Figure 1A (right panel). The peak amplitudes of hERG tail currents were recorded before and after erythromycin application. The dual temperature controller was used to control the chamber temperature. The inhibitory effect of erythromycin on the hERG potassium channel was different at RT and at PT. At RT, the percentage inhibition of hERG currents of 3, 10, 30, 100, 300, 1000, and 3000 μM erythromycin was −3.9 ± 1.3%, 1.8 ± 2.4%, 9.9.0 ± 2.9%, 15.0 ± 4.2%, 23.1 ± 6.1%, 37.0 ± 7.1%, and 60.1 ± 4.3% (*n* = 5–10), respectively (Figure 1B), while the percentage inhibition was 10.9 ± 2.0%, 20.0 ± 2.0%, 30.1 ± 2.4%, 41.9 ± 3.8%, 58.1 ± 3.4%, 89.2 ± 4.2%, and 97.1 ± 1.1% (*n* = 7) at PT (Figure 1C). Although the inhibition at RT was far from the maximum, which is not ideal for estimating IC_50_, erythromycin was not tested at 3000 μM or above because it was insoluble at this concentration. The data were fitted using the Hill equation. The IC_50_ is 1671 ± 593 μM at RT and 150 ± 26 μM at PT, respectively (Figure 1B,C). The IC_50_ at RT is approximately 11-fold higher than that at PT, suggesting that erythromycin inhibits the hERG current not only in a concentration-dependent manner but also in a temperature-dependent manner. These results are similar to those reported in the literature [21]. The inhibitory effects of 3 mM erythromycin at RT (67.8 ± 4.3%, *n* = 5) and 300 μM erythromycin at PT (58.4 ± 3.4%, *n* = 7) were obtained and are shown in Figure 1D, and are also similar to those reported in the literature [15]. These two concentrations were further used to investigate the effect of erythromycin on hERG channels influenced by temperature. 

### 2.2. Effect of Erythromycin on the Steady-State Activation of RT and PT

The steady-state activation of the ion channel is an important parameter of the voltage-gated ion channel that reflects the process of channel opening. To better understand the mechanism, we investigated the steady-state activation of hERG with erythromycin at different temperatures and then explored the kinetic properties of its action. The concentrations of 3 mM erythromycin at RT and 300 µM at PT were chosen in the study because they have similar inhibitory effects.

**Figure 2:**
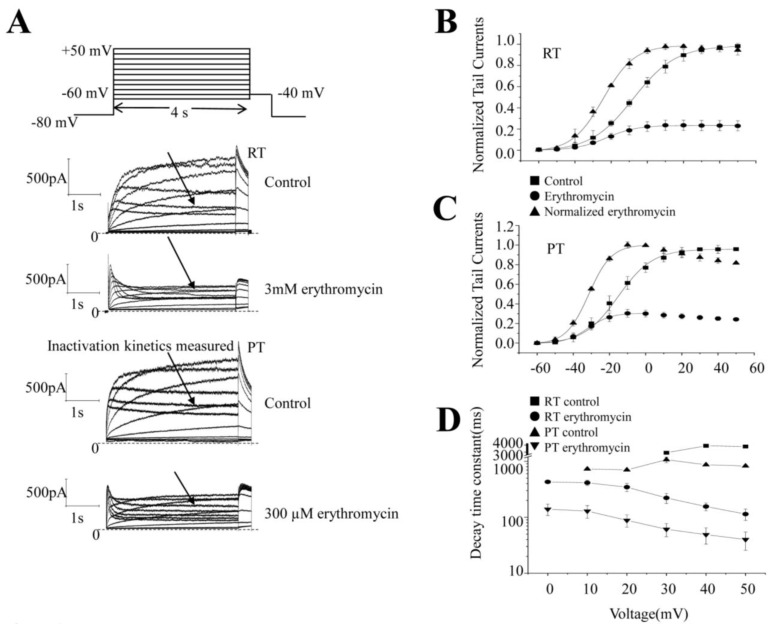
The effect of erythromycin on hERG steady-state activation. (**A**) The upper panel shows the steady-state protocol. The middle two panels are the example steady-state current traces before and after erythromycin at RT, and the bottom two panels are the current traces before and after erythromycin at RT. (**B**,**C**) ■ represents the data before erythromycin. ● represents the data after erythromycin. ▲ represents normalized data after erythromycin. (**B**) Steady-state activation before and after application of 3 mM erythromycin at RT. V_1/2_ is −7.0 ± 0.7 mV before and shifted to −25 ± 1.3 mV (*n* = 6), and the slope value is 11.0 ± 0.5 and 7.0 ± 0.6, respectively (**C**) Steady-state activation. The current–voltage curves before and after 300 μM erythromycin at PT. V_1/2_ is −17.0 ± 0.5 mV and −33.0 ± 1.6 mV at PT, *n* = 5 before and after 300 µM erythromycin. The value of the slope is 11.0 ± 0.4 and 5.0 ± 1.4, respectively. (**D**) shows the kinetics of the inactivation changes after application of erythromycin. ■ represents the data before erythromycin at RT. ● represents the data after erythromycin at RT. ▲ represents the normalized data before erythromycin at PT. ▼ represents the normalized data after erythromycin at PT. → in (**A**) represents where the data came from.

The patch clamp assay was performed at RT and PT. After the whole-cell patch clamp configuration was established, the extracellular fluid was perfused until the current amplitudes were stable. Following the protocol shown in Figure 2A, the outward currents were recorded at different voltage levels and the peak tail current amplitudes were measured before erythromycin application after the voltage levels were repolarized to −40 mV. To record erythromycin-treated currents, erythromycin was perfused for 3–5 min (Figure 2B) before the pulse stimulation program in Figure 2A was given. After the current stabilized, the current value was recorded under the same pulse stimulation program (Figure 2A). The initial data were processed by the corresponding software, the maximum current value was set to 1 at each activation voltage as the abscissa, and the percentage of the tail current to the maximum current (current density) under other different voltages was used as the ordinate to make the I-V curve.

The two steady-state activation curves are shown in Figure 2B,C at RT and PT, respectively. At RT, the ion channel opened at −40 mV and the current amplitudes increased until approximately 20–30 mV after the voltage was increased. However, the peak amplitudes were shifted to 0 mV at PT (Figure 2B). A similar shift in V_1/2_, which was previously −7.8 ± 0.4 mV and shifted to −22.4 ± 0.4 mV (*n* = 6) after the addition of erythromycin, suggests that erythromycin alters the voltage sensitivity of the channels. At PT, the V_1/2_ of steady-state activation was shifted to −17.1 ± 0.5 mV to the left compared with RT at PT. At the same time, V_1/2_ was further shifted to the left to −33.0 ± 1.6 mV (*n* = 5) after treatment with erythromycin. It was found that the slope (K) was 11.7 ± 0.5 and 8.2 ± 0.4 (*n* = 6) (Figure 2B) before and after the addition of the drug at RT, while, at PT, it was 9.5 ± 0.6 and 6.3 ± 2.4 (*n* = 5) (Figure 2C). The curve was positively shifted to the left after the addition of erythromycin (Figure 2B,C), indicating that erythromycin affected the I-V curve of hERG at RT and PT. However, the difference in the effect of erythromycin between RT and PT is small and insignificant. The leftward shift of the V_1/2_ curve by approximately 10 mV at higher temperatures is consistent with the data in the literature [22].

The inactivation kinetics of the currents were measured (Figure 2D). There were few measurable traces at RT. Their time constants (τ) were 2914.6 ms (*n* = 1) at 30 mV, 3879.5 ms (*n* = 1) at 40 mV, and 3766.7 ± 192.7 ms (*n* = 3) at 50 mV. After 3 mM erythromycin, the time constants were 413.3 ± 45.6 ms (*n* = 6) at 10 mV, 345.6 ± 57.7 ms (*n* = 6) at 20 mV, 222.0 ± 45.9 ms (*n* = 6) at 30 mV, 155.6 ± 25.0 ms (*n* = 6) at 40 mV, and 114.0 ± 26.2 ms (*n* = 6) at 50 mV. It is clearly much faster and at least 10 folds faster after the application of erythromycin. At PT, the time constants were 728.8 ms (*n* = 1) at 10 mV, 702.0 ms (*n* = 1) at 20 mV, 1068.9 ± 122.6 ms (*n* = 5) at 30 mV, 867.8 ± 32.3 ms (*n* = 5) at 40 mV, and 825.5 ± 36.8 ms (*n* = 5) at 50 mV. The time constants were 139.7 ± 32.4 ms (*n* = 5) at 10 mV, 129.0 ± 32.8 ms (*n* = 5) at 20 mV, 88.4 ± 21.8 ms (*n* = 5) at 30 mV, 48.9 ± 15.7 ms (*n* = 5) at 40 mV, and 40.1 ± 14.4 ms (*n* = 5) at 50 mV after the application of erythromycin. Comparing the time constancy at RT, the time constancy at PT is significantly faster than at the same voltage at RT. The time constants are further significantly accelerated after the application of erythromycin.

### 2.3. Effect of Erythromycin on Steady-State Inactivation of hERG Channels at RT and PT

**Figure 3:**
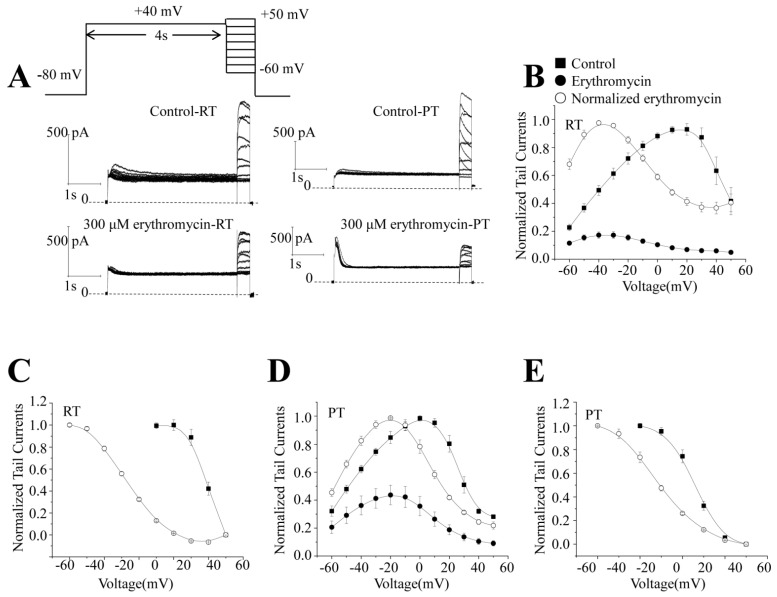
Steady-state inactivation before and after erythromycin application at RT and PT. (**A**) The upper panel shows the voltage command protocol. A 4 s prepulse to +40 mV was applied to inactivate the channels from a holding potential of −80 mV followed by test pulses from −60 to +50 mV in 10 mV increments to elicit the currents. The middle panel shows the example current traces before erythromycin application at RT and PT. The bottom panel shows the example current traces after erythromycin application at RT and PT, respectively. ■ represents the data before erythromycin. ● represents the data after erythromycin. ○ represents the normalized data after erythromycin. (**B**,**D**) show the tail peak currents following the change in voltage before and after erythromycin. (**C**,**E**) show that the steady-state inactivation by erythromycin is shifted to the left. The normalized data are fitted by the Boltzmann equation.

To better understand the effect of erythromycin on hERG potassium channels at different temperatures, we examined the effect of erythromycin on the steady-state inactivation of hERG following the pulse stimulation protocol shown in Figure 3A. After whole-cell configuration was established, the extracellular fluid was perfused until the current amplitude was stable; this typically takes 3–5 min. The example current traces are shown in Figure 3A, middle panel. After recording was completed, 300 μM erythromycin was added, and current data were recorded in Figure 3A, bottom panel. For ease of comparison, the data were normalized to their maximum current amplitudes. The average data are shown in Figure 3B,C at RT and PT, respectively. Figure 3B shows the data at RT (*n* = 10). Before erythromycin administration, it peaks at 20 mV (V_1/2_ = 39.2 ± 0.6, *n* = 10) and, after erythromycin administration, the peak shifts to the left at −40 mV (V_1/2_ = −7.3 ± 1.3, *n* = 10). At PT, Figure 3C shows that the peak tail current reaches its peak at approximately 0 mV (V_1/2_ = 25.9 ± 0.3, *n* = 5) before the addition of erythromycin and shifts to the left at −20 mV (V_1/2_ = 8.5 ± 1.0, *n* = 5) after the addition of erythromycin, which is a smaller shift than at RT (*n* = 5). The data show that the higher temperature at PT shifts the steady-state inactivation to the left. Because the concentrations of RT and PT have a similar inhibitory effect, the data also suggest a smaller effect of erythromycin on PT. However, it can also be explained that the shift caused by erythromycin is concentration-dependent, as 300 µM at PT is 10 times less than 3 mM erythromycin at RT. 

### 2.4. Effect of Erythromycin on the Onset of Inactivation of hERG Channel at RT and PT

**Figure 4:**
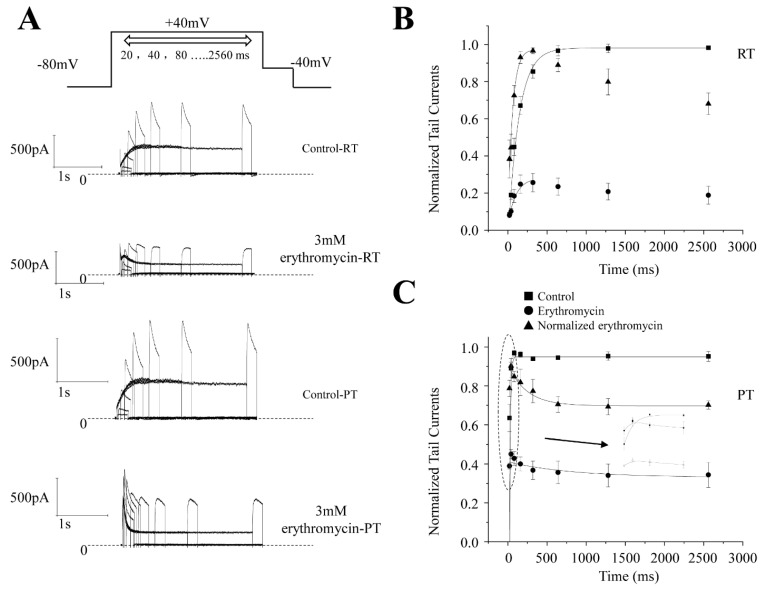
The onset of inactivation is shown. (**A**) The command voltage protocol. The holding potential was −80 mV pulsed with +40 mV depolarizing voltage for a variable duration of 20 to 2560 ms in double-duration increments. The example current traces are shown in the middle and bottom panels. Currents were recorded during repolarization to −40 mV before and after erythromycin application. (**B**,**C**) ■ represents the data before erythromycin. ● represents the data after erythromycin. ▲ represents the normalized data after erythromycin. The hERG current traces before erythromycin application at RT. The hERG current traces after application of 3 mM erythromycin at RT. (**B**) shows the recovery of the peak tail amplitudes following the different depolarizing pulse duration before and after the application of 3 mM erythromycin at RT. (**C**) shows the recovery of the peak tail amplitudes following the different depolarizing pulse durations before and after the application of 300 μM erythromycin at PT. The insert shows the detail of the change in the first four data points. Circles and → indicate where the insert has come from.

By increasing the length of the prepulse, the onset protocol was used to measure how fast the channel transitions to an inactivation state. The protocol shown in Figure 4A was used to study the effect of erythromycin on hERG channels. The peak tail currents elicited by test pulses at −40 mV were used to measure inactivation because the tail currents reflect complete recovery from the inactivation of hERG channels. 

The individual peak currents were normalized to their maximal peak tail current amplitudes and then plotted against their prepulse lengths. At RT, the peak tail current reaches its maximum at 320 ms and tends to be stable thereafter (Figure 4B, *n* = 7). Its time constancy is 144.2 ± 0.3 ms. After the addition of the compound, the maximal peak current shifts to a length of 80 ms and the time constancy is 48.8 ± 0.1 ms, significantly faster than before (*n* = 5, *p* < 0.01). Meanwhile, the maximal peak current also shifted from 80 ms prepulse length (τ = 12.6 ± 3.0 ms) to 40 ms prepulse length (time constancy cannot be adjusted because there are only two data points before the peak current, including the peak current) at PT before and after the addition of erythromycin (Figure 4C, *n* = 8). In addition to increasing the rate of inactivation, erythromycin also enhances the inhibition of tail currents after increasing the prepulse length at both temperatures. The rate of inhibition is much faster at PT than at RT.

### 2.5. Inhibitory Effect of Erythromycin on Mutations of hERG Channel at RT and PT

**Figure 5:**
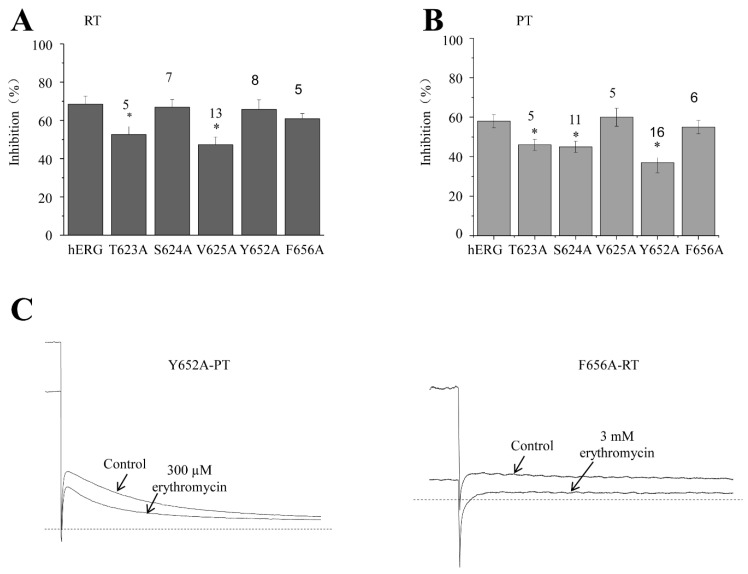
Effects of erythromycin on hERG channel mutations at RT and PT. At PT, T623A (*n* = 5), S624A (*n* = 11), S625A (*n* = 5), T652A (*n* = 16), F656A (*n* = 6). The holding potential was −80 mV, +40 mV, voltage was given, pulse stimulation lasted for 2 s and then repolarization to −40 mV took place. (**A**) Effect of erythromycin on hERG channel mutations at RT. * *p* < 0.05. (**B**) Effects of erythromycin on hERG channel mutations at PT. * *p <* 0.05. (**C**) Example tail current traces before and after erythromycin.

As we mentioned earlier, 3 mM erythromycin concentration inhibits 67.8 ± 4.3% of the hERG tail current at RT and 300 μM erythromycin inhibits 58.4 ± 3.4% of the hERG tail current at PT (Figure 1D). Further experiments revealed that the percentage inhibitions of hERG mutations T623A, S624A, V625A, Y652A, and F656A were 52.1 ± 6.0% (*n* = 5), 67.2 ± 4.1% (*n* = 7), 46.9 ± 4.2% (*n* = 13), 67.1 ± 5.0% (*n* = 8), and 61 ± 2.7% (*n* = 5), respectively, at RT (Figure 5A). Among them, the T623A and V625A mutations significantly reduced the inhibition of erythromycin, with the V625A mutation reducing more. The percentage inhibition of 300 μM erythromycin against T623A, S624A, V625A, Y652A, and F656A was 46.1 ± 2.8% (*n* = 5), 45.0 ± 2.8% (*n* = 11), 60.2 ± 4.5% (*n* = 5), 36.9 ± 5.15% (*n* = 16), and 55.2 ± 3.3% (*n* = 6), respectively, at PT (Figure 5B). Among them, T623A, S624A, and F656A mutations significantly decreased inhibition, with F656A being the strongest. It is interesting to note that the erythromycin binding sites are different at different temperatures, with the exception of T623A. In contrast to RT, where V625A has a stronger effect on RT, V625A has no significant effect on PT; while F656A has a small effect on RT, it is the strongest binding site on PT (Figure 5C). Thus, our data suggest that the critical hERG binding sites of erythromycin exhibit a remarkable shift between RT and PT.

### 2.6. Inhibitory Effect of Cisapride on hERG Potassium Channels and Mutants at RT and PT

**Figure 6:**
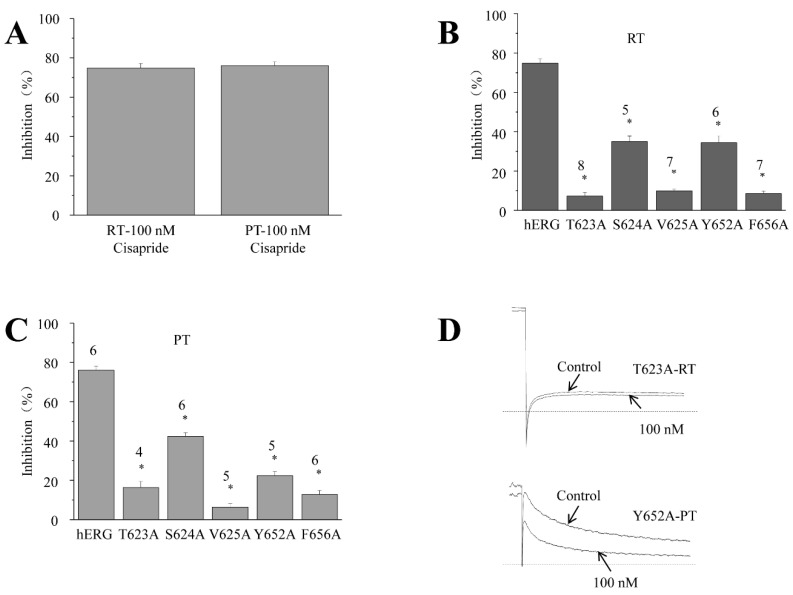
Effects of cisapride on hERG channel mutations. (**A**) The average percentage inhibition of 100 nM cisapride on hERG channels at RT (*n* = 6) and PT (*n* = 6). The holding potential was −80 mV, pulse was +40 mV voltage, pulse duration was 2 s, and then repolarization to −40 mV took place. Bar graphs show the effects of 100 nM cisapride on hERG channel mutations at RT (**B**) and PT.* *p* < 0.05. (**C**). N numbers are on top of the bars. * *p* < 0.05.(**D**) Example tail current traces before and after erythromycin.

Erythromycin is one of the few compounds that enhance their inhibitory effect on hERG when the temperature increases [15]. It is surprising to know that it has preferential binding sites to hERG at RT and PT. Further studies were prompted to determine whether other compounds that exhibit a stable inhibition of hERG at different temperatures exhibit the same phenomenon. Cisapride is a typical hERG channel blocker. It is no surprise that there is no significant difference in the inhibition of cisapride between RT and PT (Figure 6A): as the percentage inhibition of 100 nM cisapride on hERG is 75.9 ± 2.2% (*n* = 6) at RT and 75.1 ± 2.0% (*n* = 6) at PT, respectively, there is no significant difference (*p* > 0.05). Further experiments revealed that the percentage inhibition of 100 nM erythromycin against T623A, S624A, V625A, Y652A, and F656A mutations was 7.1 ± 1.9% (*n* = 8), 44.0 ± 2.8% (*n* = 5), 10.0 ± 0.9% (*n* = 7), 33.9 ± 3.6% (*n* = 6), and 8.0 ± 1.32% (*n* = 7), respectively, at RT (Figure 6B). All of them significantly reduced the inhibition of cisapride (*p* < 0.05). Among them, T623A, V625A, and F656A showed a similar strength of inhibition. Meanwhile, the inhibition of the mutation was 15.8 ± 3.1% (*n* = 4), 42.2 ± 1.8% (*n* = 6), 6.1 ± 1.7% (*n* = 5), 22.1 ± 2.1% (*n* = 5), and 12.9 ± 3.5% (*n* = 6) at PT, respectively (Figure 6C). Although the strength of inhibition changed slightly, T623A, V625A, and F656A were still the mutations that reduced cisapride inhibition the most. The current percentage inhibitions of 100 nM cisapride in five mutants at RT and PT were all significantly different from those on WT. Data from each mutant at RT and PT were compared (Figure 6B vs. Figure 6C), which showed that the patterns of the inhibition of cisapride on hERG were similar at RT and PT. Mutations S624A and Y652A have a lower effect on inhibition, while mutations T623A, V625A, and F656A show robust inhibition at both temperatures. 

## 3. Discussion

The hERG channel is such an important channel that it is listed as a biomarker for cardiac safety. The hERG channel is involved in the plateau phase of the cardiac action potential and precisely controls the length of the action potential. Any drug that blocks the hERG channel prolongs the action potential and thus prolongs the QT phase in the electro-cardiac graph, which can lead to sudden death. Many compounds have been examined to see whether they block hERG channels before being selected as drug candidates. These blocking experiments are mainly performed at room temperature, which is significantly different from physiological temperature (36–37 °C). Therefore, the question of whether compounds should be tested at physiological temperature becomes a critical question. 

Our experiments have shown that the hERG channel has many different properties at room temperature and at physiological temperature. Overall, the hERG channel at PT is faster and shifted to the left compared with the channel at RT. For example, the V_1/2_ of steady-state activation at PT is left-shifted from −17 mV at RT to −33 mV. However, the slope of steady-state activation is not significantly changed by temperature [22]. In the steady-state inactivation protocol, the voltage to activate the maximal current is also shifted from 20 mV at RT to 0 mV at PT. A similar leftward shift was also observed at the onset of the inactivation protocol [23]. A leftward shift in steady-state activation is expected for voltage-gated ion channels with a rising temperature, as the same changes have been reported at least for voltage-gated bacterial sodium channels [24] and the hERG channel itself [22,25,26]. 

It is known that the inhibitory effect of erythromycin on hERG is temperature-dependent [15]. As expected, we found that the IC_50_ was 1684 µM at RT and 150 µM at PT, suggesting that the inhibition is temperature-dependent. The result is very similar to those reported in the literature. Using different concentrations but a similar inhibitory strength, we found that erythromycin affected the gating of the hERG potassium channel. Although both steady-state activation and inactivation are shifted to the left with an increasing temperature, we notice that erythromycin appears to have a smaller effect on steady-state activation than on inactivation. After erythromycin, the V_1/2_ of the steady state is shifted to the left by 18 mV at RT vs. 16 mV at PT; the difference is 2 mV and is not statistically significant. In contrast, the maximal currents at steady-state inactivation are shifted to the left by 60 mV at RT vs. 20 mV at PT; the difference is 40 mV. Although more data are needed to support this, we suspect that the shift is dependent on erythromycin concentration rather than the strength of inhibition by erythromycin. The 16–18 mV shift in steady-state activation could be a maximal shift at a concentration near 300 µM. If this assumption is correct, then steady-state activation is very sensitive to erythromycin because 300 µM of erythromycin already maximizes the effect. The effect on steady-state activation appears to be a separate event from steady-state inactivation because the shift has such a large difference. Although activation and inactivation are linked, this should not be too surprising, as steady-state activation tends to be driven by the S4 transmembrane helix and steady-state inactivation tends to be driven by C inactivation in the filter region. S4 and the filter formed by the L5–6 helix have quite a distance between each other in the hERG crystal structure [10]. Therefore, erythromycin might have two separate binding sites close to S4 and the filter. 

The onset of inactivation occurs via the length of the voltage pulses that place the channels in the inactivated state. Both recoveries of peak tail currents are faster after the cells are treated with erythromycin. The recovery can generally be described by a double exponential curve reflecting fast and slow inactivation states, respectively. It is interesting to note that the second part of the recovery decays when following the increase in pulse length at PT. The same pattern was also observed after treatment with erythromycin at RT. It could be that a higher temperature or erythromycin interrupts the slow inactivation or decay process, as the large tail current is due to the slow decay and superfast recovery of inactivation. The interruption follows the increase in prepulse length and must overcome the recovery of inactivation and suppress the peak tail currents. In addition, erythromycin could affect the S6 water cavity domain, such as mutations in S6 of sodium channels that alter the slow inactivation [27]. 

It is surprising that the key amino acids reversed at the different temperatures (RT vs. PT). According to our data, V625 is the most important amino acid for erythromycin binding on RT. However, the V625A mutation has no effect on PT. The key amino acid is shifted to Y652, which has no effect on RT. S624 has no effect on RT, but significantly reduces the inhibition of PT. Only the inhibition of T623A remains stable. One study reported that the inhibition of Y652A was not significant [18]. The difference can be explained by temperature control. Their chamber temperature was not as well controlled as our dual-temperature controller, which automatically adjusts the temperature by directly heating the chamber. In fact, the temperature from the barrel to the chamber drops dramatically when only the solution heated in the barrel is used. 

In contrast to erythromycin, although the strength of the inhibition of cisapride by the same mutation changes significantly between RT and PT, the pattern remains the same and there is no reversal of inhibition. For T623A, S624A, V625A, Y652A, and F656A, the disruption of the inhibition of cisapride at both temperatures remains in the pattern of strong, weak, strong, weak, and strong, respectively (Figure 6B,C). The inhibition of these three mutations at both RT and PT is around or less than 20% of the inhibition of their wild type. The difference in inhibition may indicate that there is a change in hERG inhibition in most compounds [15,25], but the change is in the acceptable range in most cases. 

The reversal of hERG inhibition by erythromycin could be unique. First, as a small molecule, erythromycin is quite large, as its molecular weight (MW) is 734 Daltons and the MW of most small molecules in clinical medicine is less than 500 Daltons according to Lipinski’s rule of 5. The larger size of erythromycin could be more sensitive to the change in the structure of the hERG protein with a unique pore domain caused by temperature differences [10]. Another unique feature of erythromycin is that erythromycin has a dual structure: a “fold-out” and a “fold-in” structure. The majority is the “fold-out” structure, but the percentage of the “fold-in” structure will rise as the temperature rises [28]. This could explain the phenomenon of dramatic change as different structures bind to different sites. 

In conclusion, erythromycin is a unique molecule. It inhibits hERG channels with a leftward shift in steady-state activation and inactivation. In addition, key binding sites in the pore domain changed dramatically at different temperatures (RT and PT). These features could be a major reason for the dramatic increase in inhibitory strength from RT to PT. Therefore, our data suggest that erythromycin is unique and that the shift in hERG inhibition may not apply to other compounds. In the early stages of drug discovery, the goal is to find suboptimal structures or lead compounds. The purpose is to save time and money at this stage. Our data strongly suggest that studying the strength of the inhibition of the hERG channel by compounds at RT is a good fit, even it is not perfect.

## 4. Materials and Methods

### 4.1. Preparation of Erythromycin and Cisapride

The 100% pure erythromycin (Prefa, Chengdu, China) was dissolved in DMSO (Aladdin, Shanghai, China) as a storage solution and stored in the refrigerator at 4 °C before the experiment. On the day of the experiment, erythromycin was added to the extracellular solution at the concentration required for the assay. Cisapride (Sigma, St. Louis, MO, USA) at a concentration of 9 mM as stock solution was diluted to the required concentration for the patch clamp test.

### 4.2. Cell-Line Preparation

HERG stable cell line and Human Embryonic Kidney (HEK-293) cells were cultured in a solution containing 10% (*v*/*v*) fetal bovine serum (FBS), 1% (*v*/*v*) P/S (100U Penicillin and 0.1 mg/mL streptomycin). Additional 100 μg/mL G418 were added to culture hERG stable cell line. Cells were passaged and transiently transfected with 1 µg WT and T623A, S624A, S625A, T652A, and F656A mutations using transient transfection reagents (Attractene Transfection Reagent, Qiagen, Hilden, Germany), while CD8 cDNA was co-transfected. The successfully transfected cells were identified by labeling with CD8-specific antibody-coated microspheres (Dynal, Oslo, Norway).

### 4.3. Electrophysiological Recordings

Erythromycin samples were weighed accurately according to their molecular weight. They were then dissolved in DMSO to prepare a 3 M highly concentrated mother liquor, which was stored at −80 °C. On the day of the whole-cell patch clamp experiment, the highly concentrated mother liquor was added into DMSO to prepare 900, 300, 90, 30, 9, 3, and 0.9 mM mother liquor, respectively. Finally, 100 μL of the corresponding mother liquor was added into 30 mL of extracellular solution to prepare the medicinal liquid with a concentration gradient of 3000, 1000, 300, 100, 30, 10, and 3 μM for practical use.

The hERG channel currents were recorded using a whole-cell patch clamp. The current wave curves were recorded using the PC 505B (Warner) clamp amplifier at room temperature (RT, 20–22 °C ) and physiological temperature (PT). The PT refers to 35 °C ± 2 °C in our study. At PT, the temperature of the bath solution was maintained by a combination of in-line solution, preheater, chamber, heater, feedback temperature controller, and thermistor probe in the bath (TC-44B dual automatic temperature controller, Warner instrument, Hamden, CT, USA). hERG channel currents were recorded using a whole-cell patch clamp filled with a potassium aspartate solution (140 mM potassium aspartate, 10 mM HEPES, 5 mM Mg ATP, 2 mM MgCl_2_, 11 mM EGTA; pH 7.3, adjusted with KOH). The bath solution contained NaCl, 136 mM; KCl, 4 mM; CaCl_2_, 1.8 mM; MgCl_2_, 1 mM; glucose, 10 mM and HEPES, 10 mM; pH 7.3 with NaOH. 

Erythromycin samples were weighed accurately according to their molecular weight. They were then dissolved in DMSO to prepare a 3 M highly concentrated mother liquor, which was stored at −80 °C. On the day of the whole-cell patch clamp experiment, the highly concentrated mother liquor was added into DMSO to prepare 900, 300, 90, 30, 9, 3, and 0.9 mM mother liquor, respectively. Finally, 100 μL of the corresponding mother liquor was added into 30 mL of extracellular solution to prepare the medicinal liquid with a concentration gradient of 3000, 1000, 300, 100, 30, 10, and 3 μM for practical use.

The protocol used to study IC_50_ is shown in Figure 1A, top left, as follows: current recordings are triggered with a depolarizing step to +40 mV for 2 s from a holding voltage of −80 mV, and then a repolarizing step to −40 mV is used to record hERG tail currents.

### 4.4. Data Analysis

All test data were expressed as mean ± S.E.M. Clampfit, Execl 2010, and Origin 8.0 software were used for statistical analysis and image processing. The Boltzmann equation was used for steady-state activation and inactivation; the Hill equation was used to calculate IC_50_; other fitting functions are mentioned in the results section. Statistical analysis was performed by T-test. The difference, *p* < 0.05, was significant.

## Data Availability

The data that support the findings of this study are available from the corresponding author upon reasonable request.
